# Concurrent analysis of choice and control in childbirth

**DOI:** 10.1186/1471-2393-11-40

**Published:** 2011-06-01

**Authors:** Austyn Snowden, Colin Martin, Julie Jomeen, Caroline Hollins Martin

**Affiliations:** 1 Research Fellow in Psychological Care and Therapies and Lecturer in Mental Health Nursing. School of Health, Nursing and Midwifery, University of the West of Scotland, Paisley Campus, Paisley, PA1 2BE, Scotland, UK; 2 Professor of Mental Health. School of Health, Nursing and Midwifery, University of the West of Scotland, Ayr Campus, Beech Grove, KA8 0SR , Ayr, Scotland, UK; 3 Associate Dean Research and Scholarship. Faculty of Health and Social Care, University of Hull, Cottingham Road, Hull, HU6 7RX , England, UK; 4 Senior Lecturer, Department of Community, Women and Children's Health, The School of Health, Glasgow Caledonian University, G4 OBA , Scotland, UK

**Keywords:** Choice, control, pregnancy, narrative, qualitative, generalisable, concurrent analysis

## Abstract

**Background:**

This paper reports original research on choice and control in childbirth. Eight women were interviewed as part of a wider investigation into locus of control in women with pre-labour rupture of membranes at term (PROM) [1].

**Methods:**

The following study uses concurrent analysis to sample and analyse narrative aspects of relevant literature along with these interviews in order to synthesise a generalisable analysis of the pertinent issues. The original PROM study had found that women experienced a higher degree of control in hospital, a finding that appeared at odds with contemporary notions of choice. However, this paper contextualises this finding by presenting narratives that lucidly subscribe to the dominant discourse of hospital as the safest place to give birth, under the premise of assuring a live healthy baby irrespective of their management type.

**Results:**

This complex narrative is composed of the following themes: 'perceiving risk', 'being prepared', 'reflecting on experience', maintaining control' and relinquishing control'. These themes are constructed within and around the medical, foetocentric, risk averse cultural context. Primary data are presented throughout to show the origins and interconnected nature of these themes.

**Conclusions:**

Within this context it is clear that there is a highly valued role for competent health professionals that respect, understand and are capable of facilitating genuine choice for women.

## Background

The concept of choice as an integral aspect of contemporary heath care policy is now relatively well embedded. Current maternity policy [2,3] advocates choice and control for childbearing women equating these elements to both a better quality of experience and improved outcomes. Choice, however, would seem less straightforward than policy assumes. Choice is an act, which requires intimate connections between reason and rationality, a weighing up of risks and benefits and an ordering of preferences based on their utility [4]. It is feasible to suggest that because outcomes during pregnancy and birth are uncertain, that women may consider choice not only to be about desires for a certain birth experience but also a gamble.

Whilst women appear to desire choice in maternity care, it is important to recognise that women make choices for a whole set of often complex reasons. However, we also know that choice is constructed through pervading belief systems and resources [5]. Perceptions of risk, defined predominantly by medical experts, have mapped out what a 'responsible' decision should be and to question or ignore those definitions of risk is to be labelled a 'bad mother' [6]. This is lucidly illustrated by findings, which demonstrate that the majority of women, continue to cite hospital 'as the best place to give birth' and make choices accordingly [7]. Safety is a key issue in maternity care and despite the fact that childbirth has never been safer in the developed world, in terms of mortality, and the safety of labour and birth at home has been established [8], fear of birth amongst mothers remains [9,10].

Evidence seems to suggest that the constructs of choice and control are intimately connected for women with regard to pregnancy and their childbirth experience. The opportunity for greater choice over care allows more involvement with decision-making and impacts on a woman's feelings of control, with control being significant in terms of women's satisfaction with their birth experience. Women who choose home birth often claim to do so under a premise of retaining control [11,12]. For example a randomised control trial (RCT) by Martin & Jomeen (2004) [1] investigated home versus hospital management of women with a prelabour rupture of membranes (PROM). They found women in the hospital group displayed higher internal locus of control scores than those in the home group at the onset of labour or prior to induction of labour. This suggests that being in hospital facilitated greater feelings of women's personal control at that point in time than being at home, a finding which seemed counter-intuitive in light of other evidence.

In order to elucidate this finding qualitative interview data was also collected from some of the women who had already consented to and been part of the RCT. Separate ethical consent, for this aspect of the study was obtained from the Local Research Ethics Committee. Women were selected on the basis of being involved in the PROM trial, with babies no older than a year. Initial contact was by letter and then telephone, thirteen women initially responded but only nine were available to be interviewed within the time scale of the study. The interviews adopted a conversational approach with the aim of generating narrative data. Interviews were arranged at the convenience of the women and took place in either home or hospital settings dependant on the woman's preference. All interviews were tape recorded and transcribed verbatim. The content of the interviews was determined initially by the women's experience of being part of the PROM study, however women actually narrated much broader stories of their recent and previous birth experiences to explain their feelings. Narrative themes were then identified using a comparative process and composite stories representative of the women's experiences were developed.

One potential interpretation of the PROM study findings was that control was being conceptualised differently for the hospitalised women and linked to the 'safety net' of the hospital. A first reading of the qualitative data appeared to potentially contradict that interpretation. That is, women appeared to tell stories which reflected their appreciation of being given the choice to go home, of being more relaxed at home and consequently experiencing heightened perceptions of control. However the interview narrative provided enlightening reasons as to the apparently contradictory findings. Women's narrative despite displaying a positive attitude and experience in relation to home management, illustrated the embedded nature of the medical model of childbirth and the pervasive nature of the construct of risk. This is turn led to a subsequent subordination of women's own needs to those of the baby wherever necessary. Narratives of those women who stayed in hospital, felt a sense of control because they perceived an assured safety of their baby through staying in hospital. In contrast those women who went home, despite being given permission, were always troubled about whether being at home would compromise the safety of their baby and hence felt less in control at the onset of labour. In relation to choice and control women are simultaneously assigned active and passive roles, Despite a desire to articulate their wishes, a lack of ownership of their pregnant and labouring body's does not enable them to do so. This paper seeks to enhance and extend the seemingly disparate findings of the quantitative and qualitative aspects of the PROM study.

## Methods

Concurrent analysis (CA) is a new methodology designed with the purpose of increasing the generalisability of qualitative findings. Whilst this aim is not new, CA differs from recent approaches to qualitative synthesis [13-15] by integrating interview data specifically gathered by the researcher. This ensures the product of analysis remains focused on answering a specific research question but extends the generalisability of the findings by conducting the literature review as part of the data collecting process.

The thinking behind this is developed in detail elsewhere [16] and demonstrated in practice within a constructivist grounded theory methodology [17]. In brief CA removes any delineation between similarly constructed data. It treats aspects of the literature *as *primary data where the focus of the literature is equivalent to other primary data under study. For example if the researchers ask questions about choice in childbirth and other researchers have published studies about the same topic then these data can be treated as conceptually equivalent.

The need for CA arose from recognition of 2 separate but interrelated positivist remnants in the qualitative literature:

1. Grounded theory retains a 'before or after' argument about when to engage with literature. This argument is grounded in issues of bias, yet bias is irrelevant to a constructivist explanation of a social process.

2. Metasynthesis potentially excludes important but 'low level' qualitative research such as case studies. These important studies are rated as low level as an artefact of quantitative hierarchies. These hierarchies may be irrelevant to qualitative questions, where a case study may be the best and most appropriate methodology. Excellent research may therefore be excluded erroneously from even the best metasynthesis.

Concurrent Analysis therefore synthesises primarily through *inclusion *of all relevant material. Exclusion criteria are not based upon methodological type but methodological *quality*. In this study, where narrative or description was reported first hand and related to issues of choice in childbirth this data was considered appropriate for concurrent analysis. Narrative and descriptive data existed in the literature comparable with the interview data. No distinction was subsequently made in ascertaining themes. Thematic analysis involved first highlighting the relevant sections of text or interview related to the issues of choice and control in childbirth. The next part of the analysis involved extracting codes (meaning units) from these data. Codes were then compared with each other to ascertain contextual and conceptual similarities and differences. For example categories of thoughts or behaviours could be indentified related to feelings about perceived risk in childbirth. These codes and categories were then compared with each other to ascertain depth and breadth of the decontextualised issues.

Despite the inclusive nature of the method it was important to ensure the analytic process remained robust. To this end only peer reviewed high quality literature was selected for analysis. It is recognised that global criteria for judging quality of qualitative research are problematic due to the differing philosophies underpinning the differing methodologies under this umbrella. There is wide agreement however that qualitative research should be ethical, important, clearly articulated and use appropriate, rigorous methods [18]. The papers analysed here have met these criteria. In the original PROM study 8 interviews were undertaken with women regarding their experiences of home and hospital approaching childbirth. The focus of the interviews was on exploration of their feelings using a narrative analytic approach. The literature was searched for comparable narrative exemplars (Table 1).

**Table 1:** Literature search, inclusion and exclusion criteria

Search Criteria		Returns
CINAHL	In abstract: (Labour OR labour OR childbirth) AND pregnan* AND narrative AND choice AND control	41 articles
Pubmed	(Labour OR labour OR childbirth) AND pregnan* AND narrative AND choice AND control	55 articles
ProQuest	(Labour OR labour OR childbirth) AND pregnan* AND narrative	20 articles

A further source of information was to follow leads from the reference lists of all the above literature to ensure as few as possible pertinent sources of information were missed.

Papers were included for review if they entailed narrative or description reported first hand, related to issues of choice in childbirth, and grounded in experience of that process.

Papers were included if they were methodologically coherent. This judgement was made based on Cohen and Crabtree's quality criteria (18) for qualitative research. All papers included were adjudged to be ethical, important, clearly articulated and used appropriate, rigorous methods.

Papers were excluded if they did not contain primary qualitative data from original research, if they did not meet the quality criteria, or if they were reported in language other than English.

Table 2 presents summary data from papers that contained relevant first hand narrative.

**Table 2:** Literature summary

Author(s)	Sample	Methodology
Munro et al, 2009 [20]	17 primiparous women in British Columbia, Canada.	Semi structured interviews: exploratory qualitative study
Fenwick et al, 2008[10]	14 women who had requested a caesarean section in their first pregnancy in Australia	Telephone interviews: exploratory descriptive approach:
Kennedy et al, 2009[21]	234 women during the postpartum period in US	Qualitative interviews: narrative and thematic analysis
McCourt &Pearce, 2000 [22]	20 UK women receiving different models of maternity care	Semi-structured narrative interviews at 6 months postnatal
Houvouras, 2006[23]	15 postpartum women in US	Active and feminist interviews: Constructivist grounded theory
Parry, 2006 [24]	Personal reflection and 8 women in Canada, 1 in US (7 pregnant, 2 postpartum)	Personal ethnography and interviews: narrative presentation
Crossley, 2007[25]	Single case study in UK	Personal reflection, unfolding narrative: exploratory qualitative design
Shaw, 2007 [26]	Critical response to Crossley (2007)	Personal reflection grounded in doctoral thesis data
Stokhill, 2007 [27]	Critical response to Crossley (2007)	Personal reflection grounded in autoethnography
Namey & Lyerly, 2010 [28]	72 US women who as part of a larger study had spontaneously mentioned control	Semi structured interviews: Concept analysis
Hall & Holloway, 1998[29]	9 UK women who chose to give birth in water	In depth interviews analysed using grounded theory
Walker. 2005 [30]	32 UK women who had delivered in a midwife led care unit	In depth focused interviews, analysed using grounded theory
Viisainen, 2001[31]	21 women and 12 partners of women who had planned a home birth in Finland	Semi structured interviews, narrative and thematic analysis
Viisainen, 2000 [32]	21 women and 12 partners of women who had planned a home birth in Finland	Semi structured interviews, narrative and thematic analysis
Morison et al, 1998[8]	10 couples who had a home birth in Australia	Interviews and homebirth video observation: phenomenological approach
Morison et al, 1999[33]		
Kontoyannis &Katsetos, 2008[34]	12 women who had experienced planned home birth in Greece.	Semi structured interviews: phenomenology
Kennedy &Shannon, 2004 [35]	Purposive sample of 14 midwives in US	Interview data: Narrative analysis
Lynn Clark et al, 2003[36]	Childbearing women in US, Scandinavia, Middle East, China and Tonga (n = 100)	Secondary analysis of narrative transcripts
Lundgren & Dahlberg, 1998[37]	Nine women, four primiparous and five multiparous who were two to four days post delivery.	Interview data: Narrative analysis with phenomenological interpretation of meaning
Halldorsdottir & Karlsdottir, 1996[38]	14 postnatal women in Iceland	Interactive interviews: Phenomenology
Maher, 2008[39]	10 postnatal women between 3 and 12 months in Australia	Semi-structured interviews: Narrative based approach
McCallum & Reis, 2005[40]	26 women admitted for childbirth in Brazil	Participant observation and semi structured interviews: ethnographic and narrative analysis
Liamputtong, 2009 [41]	15 middle class mothers, 15 lower class mothers in Thailand	Semi structured interviews: phenomenological thematic analysis

The local NHS ethics committee approved the qualitative aspect of the PROM study as an extension to the original PROM study [1,19]. Permission was granted by the Hull and East Riding Local Research Ethics committee.

## Results

**Figure 1:**
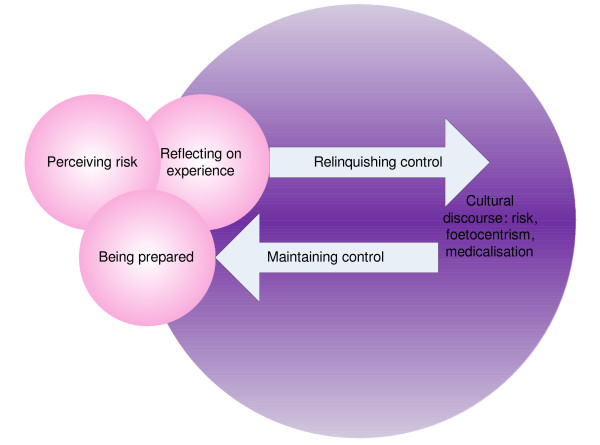
**Locus of control: Thematic analysis of factors impacting on choice in childbirth**.

All the data were subject to constant comparison in order to facilitate thematic analysis [42]. NVivo 8 was used in the early part of this process in order to maintain oversight of the burgeoning codes and memos. The model in figure 1 was developed and refined throughout a series of discussions between all authors [43]. This was an iterative but reflexive process [44] aimed at parsimony whilst retaining overt connection to the primary data. Whilst clearly utilising aspects of grounded theory within the analytic process the product is not claimed to be a grounded theory as the inclusive nature of the sampling meant that ethnographic, phenomenological and narrative analytic data were included where possible.

Figure 1 illustrates the interconnected nature of the final themes emerging in this study. The three small circles represent the dominant themes present in the individual women: 'perceiving risk', 'reflecting on experience' and 'being prepared'. The arrows describe the issues pertinent to maintaining or relinquishing control during childbirth, and the large circle represents the context, the dominant discourse. For ease of discussion the individual themes are considered separately in the first instance to illustrate how they arose from the data. Detailed exemplars accompany this discussion.

### Perceiving risk (Table 3)

**Table 3:** Perceiving risk

Examples from the interviews	Examples from the literature
When you're not living far from the hospital, you know you can always go in, it's only a short car ride.(Interview 3)	[In hospital] the safety time limit from labour ward to operating theatre is 10 minutes. I live so close I could make it (Viisainen 2000, p801)
I knew I wasn't that far away from the hospital um(p..). I weren't panicky as such, we talked about it and he you know said 'it's totally up to you, if you want to go home, we'll go home, as soon as you want to go back we'll just get in the car and go back.(Interview 1)	Mia didn't tell them anything [about the plan, during ultrasound]. We thought that if they had known about our home birth plan we could not be sure they would give us honest information, they would start finding things. [We had this before. I]f you want something alternative they [health staff] start finding things. (Viisainen 2000, p807)
I want to know that I'm in a place where everything's on hand, if anything happens whatsoever, everything's there you know...[i]f the contractions had come on really really fast at home and that had happened that would have panicked me. (Interview 8)	There is risk in everything you do and to me, having a caesarean section presented me with less risk than the vaginal. I felt I was bypassing the risk and so did my doctor' (Fenwick et al. 2008, p398)
With Emily, I didn't start in labour at all and I expected to either start in labour or be induced but I did expect to be kept in. I thought that that normal ... I think I would have preferred to stay in if it was my first one I think I would have felt a bit more worried.(Interview 3)	... the great danger (of intervention) is at the hospital. The immense danger comes when you start interfering with something as natural as giving birth.' (Nepheli) (Kontoyannis & Katsetos 2008, p46)
	Giving birth in hospital is like making love in a railway station. (Viisainen 2001, p1114)

Within the interviews hospital was firmly perceived as the right place to be to have a baby. Any deviation may be acceptable but the women needed to know that they can return to the cocoon of safety at any time. These women's stories suggest that they are happy to own the responsibility for their pregnancies up to a certain point, but they also identify a time when it is time to relinquish control and return the responsibility for childbirth back to 'the professional'. For all the women in the interviews stories revolved around living in close proximity to the hospital enabling them to return quickly if they needed the security that they perceive the hospital provides. In the women's mind it seemed to reduce the element of risk potentially involved in choosing to go home.

The broader literature is not so clear cut, and whilst perceptions that support the notion of childbirth as risky [10] and hospital as a place of safety [32] are evident, there is substantial evidence of the opposite perception. Hospitals are construed by some [31,34] as unsafe. This is because they are for sick people and pregnancy is not an illness but a natural process [33].

Although many women are committed to childbirth as normal and natural their priority is the delivery of a safe and healthy baby [45]. For some women this involved choosing an elective caesarean without clinical indication [10]. The literature provides wider accounts of this foetocentrism [24] leading women to rely on the expert to tell them all is well. Childbirth has become an axis of self-doubt [46] with many women having lost confidence in their ability to birth without intervention and consequently women rely on expertise to validate 'safe choices' as evidenced by the woman whose choice of an elective section was validated by her doctor [[10], p398]

Safety is a therefore a key narrative discourse that pervades. It is a multifaceted issue grounded in issues of risk and harm to both mother and baby, and also in these litigious times to vulnerable health care providers, often defined through engagement with the medical system. Even those women who chose a home birth engaged with the medical system, if only to the extent of ensuring the safety of their home birth plan. Viisainen [32] argues that describing home birth as 'risky' is social coercion directed at compliance with the system and the literature clearly evidences examples of women's need to defend their decisions when they fail to comply [32,33].

### Reflecting on experience (Table 4)

**Table 4:** Reflecting on Experience

Interviews	literature
I'd definitely choose to come in [to hospital] when I was ready.... I mean most first time parents I think probably want to stay in where as people that have been there before probably want to go home. (Interview 5)	The amount of choice and control I felt I had in each of these births varied, and not as might be expected in the form of a positive correlation between experience and confidence; my fourth birth was in many respects as 'taken over' and managed as the first. (Stockill 2007, p574)
I'd probably come home [next time], because you know what's going on then don't you. People feel more comfortable in their own homes. I think that's the difference when it's your first one and you're young, you don't know what's coming erm, probably with your second one you'd be alright to go home, you'd know when to say 'I think now I need to go back for the pain' or whatever. Next time I would come home.(Interview 4)	Maybe I was more courageous with the fourth because the previous one had been a fast and good birth...(Viisainen 2001, p1116)
There's a lot of pressure especially when it's your second baby I think you don't realise how in a sense it's easier to have a first baby, than it is for subsequent babies because you're torn between wanting to do your best for the baby you're having and the one that's left at home its quite a difficult scenario isn't it?(Interview 5)	You see things totally different afterwards, you have another way of understanding, you accept things differently, you become stronger, you can cope with things better than before, before petty details could ruin life, and now you just shake it off your shoulders, you don't become another personality, but you mature and become a stronger personality, when you've had a baby and have gone through that pain. I think that is the purpose of it, what the meaning of life is. I think it is to protect our children, to be stronger, a way of managing everyday life and become stronger, and that it is a life from your own flesh and blood and that too helps you to go through the delivery. (Interview 2) (Lundgren & Dahlberg, 1998)
	...we were talking for some time but because I had bad experience with N I was scared (McCourt and Pearce 2000, p150)

The narrative theme identified here suggested that previous experience impacted on women's decision making. Although the women interviewed generally welcomed the choice to either go home or stay in hospital, they all felt that they could make these decisions better in the context of their previous experience. This is unsurprising, but complex.

Weaver [47] argues that those expecting their first babies are bound by the psychological consequences of the medical model on many sides. She believes it restricts their ability to listen to their own expertise about their own bodies, a concept which seems reinforced by the interview stories and the women's claims that they would know how labour *feels *next time. Their expertise in the knowledge and ownership of their bodies gained from experience would allow them to more confidently make the decision to stay at home in future births. The quotes from the literature support the difficulties of not knowing what to expect in a first labour and birth which also potentially limits feelings of control and hence choice of birthplace. The literature further illustrates how a good outcome endorses the choices made and influences choices for the future. Lundgren and Dahlberg [37] highlight the potentially transformational nature of this. Conversely, the woman in McCourt & Pearce's study )[22] explained how a previous bad experience influenced both her expectation of her subsequent labour and birth but also her choices of care and caregiver.

The stories surrounding first pregnancies imply their experience would impact on choices for the future. Predominantly women welcomed the choice offered, but to a degree they also would prefer to have being told 'this is what happens'. With choice making comes a certain amount of responsibility for the subsequent outcome and some women find that uncomfortable. The narrative examples from the literature demonstrates that 'real choice' is about more than a desire for a certain experience but also knowledge of the options available and the ability to weigh up the costs and benefits. Whilst for some this is easier in the face of experience, women do acknowledge how each experience may feel different and must be judged by its own merit

### Being prepared (Table 5)

**Table 5:** Being prepared

*Interviews*	Literature
*umm I think its hard when its your first child you don't really know what to expect and like you do your birth plan and it just goes out of the window because you don't really know what is going to happen until you get there (Interview 2)*	I had several disagreements with my doctor concerning specific issues that finally assisted my decision on a home birth. For instance he said that shaving, enema and episiotomy will definitely take place because I am a first-time mum and it is very important not to lose the flexibility of my pelvic floor. (Kontoyannis & Katsetos 2008, p47)
*I mean you can have a choice of where you want to have your baby you know. Like at home or whatever and I was like there's no way I want the baby at home I want to be in hospital. I want to know that I'm in a place where everything's on hand, if anything happens whatsoever, everything's there you know.(Interview 3)*	She had a second child and had it planned right, so like I called her up and said, 'When's the baby due?,' and she was like, 'Oh you know, like July 1st at 3:15.' And I'm like 'What?,' and she's, 'Oh we're planning it this time. If I couldn't do it the first time I'm not doing it the second time.' And starting from then, I sort of went, 'Oh, what a civilized way of doing it. (Munro et al. 2009, p376)
*During the nine months of being pregnant I was always a bit unsure because there is a story going round at work that somebody had been paralysed after having [epidural] and its always in the back of your mind something like that...and er my friend she had a baby a month before I had [mine] and she had an epidural and after I heard the story it put me at ease having it so I thought yes I will and once I was in the pain I thought (laughs) I don't want any medals (Interview 7)*.	What struck me during my research was how difficult I personally would find it (with my limited understanding of many medical interventions related to childbirth) to exercise choice about birth if it meant being assertive with health care professionals who were not encouraging and who might set my choice against the 'needs', 'safety' and 'well-being' of the unborn child. (Shaw 2007, p566)

Clearly linked to the previous theme, most women have some sort of plan as to how and where they hope to give birth. There are a range of decisions and unfolding events that remain unknown. As a consequence interviewee 2 expressed no sense of surprise or dismay when the birth plan 'just goes out of the window'. By contrast an interviewee in Viisainen's [32] study took a different approach to this lack of knowledge by applying critical appraisal to the medical literature in order to better inform her position. Shaw [26] goes on to point out that even this approach was not enough to generate enough confidence in an independent plan. That is, her knowledge did not equip her with enough information and/or expertise to confidently challenge any discouraging medical view, particularly when the stakes are so high and expressed in terms of the primacy of the unborn child. Even knowledge generated through professional experience is not enough to ensure the experience unfolds as anticipated [39]. Whilst women may engage with expert accounts this still fails to translate to 'expertise' and the associated power to enact desire and make true choices.

Jomeen [49] has demonstrated how women are often surrounded by 'horror story' accounts of giving birth, as in the example of the epidural (interview 7), but positive birth stories can also be influential in women's' decision making around labour and birth, hence a 'story' of disaster is assuaged with a story of a happy ending. For Kontoyannis and Katsetos [34] the knowledge underpinning decision making is provided unwittingly by the doctor's 'horror story' describing the routine episiotomy, shave and enema prior to a planned hospital birth. Consequently, the woman chooses home.

The risk theme highlighted earlier also features in regard to being prepared. The priority for the woman in interview 3 is for a healthy baby, and she makes it clear her choice is to this end. Her plan is to have 'everything on hand', and this is a common aspiration. The use of planned caesareans and inductions are increasing, assumed in part to be due to doctor/patient preference [50] and although little is known about the extent to women are requesting planned interventions, they are often framed as life choices [51]. Munro et al.'s [20] interviewee describes this as 'civilised'. Mander [52] highlights how contemporary society and the media in reporting on those celebrities who request birth by caesarean and have their requests granted, despite no obvious medical indication, act as a significant influence to women. Such high profile events reinforce birth as inherently risky and promote a caesarean as the way to avoid the risk, in addition to making it the 'trendy choice'.

### Maintaining control (Table 6)

**Table 6:** maintaining control

Interviews	literature
...[R]eally I didn't want anything anyway except gas and air but when I got there I was thinking mmm shall I have an epidural but she said well you need to decide I mean you'll probably be a while just try the gas and air and I said all right then and I didn't need anything else anyway (Interview 8)	I found myself incredible, because I saw that woman screaming, creating a huge scene, and I just stayed normal. I was calm. And so, I was like, when the pain came strong I was ... after each one I would doze off, you know. It was really no problem, I bore it all really well. (McCallum & Reis, 2005)
[I]ts amazing how your body adjusts and you get used to it every stage that you really cope its amazing after Syntocinon, you see the pain is very intense very very quickly so who wouldn't want a break but then again...[i]t was an absolutely gorgeous experience without pain relief it was and I had loads of feelings as well afterwards which I suppose I didn't know about first time around I was just legs up stitches where I didn't know. (Interview 5)	I didn't want to be there alone ... and it helped to have someone to actually just talk to, like as a friend. That turned me around right there. I started feeling more in control and I started thinking positively"(Namey and Lyerly 2010, p773).
I think they were threatening caesarean at one point and I said oh I don't want to have a caesarean I want to have my baby naturally and then I think he decided to put in an appearance and she said you know go on push then and yes it was just like as if by magic it all came together yes it was great (Interview 7)	The experience of childbirth made me grow up a lot. It really did. I've learned a lot about my capacity. When I thought I was just too tired to push any more I found another 15 minutes worth of it. I just learned I have a lot more strength than I thought I did. Childbirth brought me more in tune with my body because I know what my capacities are: My mental capacity, my strength. I just know I could do a lot more than I thought I could. (Lynn Clark et al. 2003 p147)

Control in this study describes the degree a person is or perceives themselves to be in charge of their own experiences. The literature surrounding choice in childbirth intimates that the value of choice is related to the positive feelings of control it imbues in women [49]. The findings here support that claim. Control means different things to different women but all the evidence points to positive outcomes where control is perceived to have been maintained, irrespective of the birth environment that is chosen to facilitate that.

There is a strong connection between how women construct the concept of 'natural' and the perception that control has been maintained. Like safety, the construct of 'natural' has a subjective component. It often equates with minimal intervention, but it is multidimensional. For example there is often a mystical or spiritual component. Interviewee 7 invokes 'magic' as a turn of phrase to reflect on the avoidance of caesarean and the concomitant maintenance of control. This spiritual connection is more explicitly religious in much of the wider literature [40], where faith provides a further expression of strength and control linked to the construct of 'natural'.

There is evidence throughout of the positive consequences of maintaining control. Lynn Clark et al. [36] describe childbirth bringing the interviewee 'more in tune', having a 'lot more strength than I thought I did'. These quotes reinforce the power of the female body to give birth, which intrinsically links to feelings of personal control. Being in control enables women to focus on the birth, and pain for some is seen as a facilitating embodied experience. For example interview 5 describes childbirth without pain relief as an 'absolutely gorgeous experience'. [28] equate being in 'charge of the pain' to a sense of control. Interview 8 describes how she was helped to retain control by being given a gentle rejoinder to try with just the gas and air whilst edging towards asking for an epidural. Just having gas and air was clearly this interviewee's expressed wish beforehand and this is the outcome. There is wider evidence these feelings of empowerment can be facilitated by the attending health professionals, which is clearly highly valued. Hall and Holloway [29] and Halldorsdottir & Karlsdottir [38] articulate this theme in the wider literature by providing positive example of the impact of the competent health professionals on their interviewees.

### Relinquishing control (Table 7)

**Table 7:** relinquishing control

Interviews	Literature
...I just thought what's the point, I thought just get the baby out I mean I'd been in labour for about twelve hours they said he was getting distressed and I thought just get him out you know and make sure he's OK.(Interview 3)	So, with my first baby I couldn't be on top of the situation in the hospital and I did lose control. I just couldn't be in a fighting frame of mind all the time.' (Kontoyannis and Katsetos 2008, p46)
I was... determined to try and stay at home for as long as I could, until I (laughs) couldn't take it any longer (Interview 7)	I was afraid that the baby might be in danger, but I felt confident in the doctor. He possessed knowledge of getting the baby out by suction, so I trusted that he would be able to help me. (Liamputtong, 2004)
At that stage you know in a way, you're glad to get to the hospital anyway and put yourself in their hands, but I didn't know how long it was going to be. I wasn't really sure,... it did get to the point when the pains really did get quite bad and I thought I'd like to go in now you know and put myself in the midwives hands, which is what I did. I don't think there was any doubt that I would end up in hospital you know, for the safety net I suppose. (Interview 6)	I allowed myself to sit back on the bed and breathe a secret sigh of relief. One of the midwives arranged for some lunch to be brought to me, and I sat and ate it slowly, then leaned back and closed my eyes. I didn't sleep, but just felt the sense of relief flood over me - at last, someone was taking things out of my hands and I could allow myself to 'let things go', if only because that's what the medical staff were telling me I had to do. (Crossley 2007, p553)
	...my obstetrician said 'this is what's going on' and umm, look I trusted her absolutely. We had talked about the alternatives [before] and I just totally trusted her.(Maher 2008, p134)

Relinquishing control is sometimes necessary. At this point Shaw describes challenging medical knowledge not as a 'sign of strength and Amazonian empowerment, but more a sign of stupidity and weakness' [p559]. There is however a narrative of failure that permeates some of the responses evidenced at this point and many of the women articulate the difficulty of maintaining control in fighting terms (interviews 3 and 7, [34,25] described this battle as a function of uncertainty grounded in being an unequal participant in a medical world. However, this feeling of failure is not ubiquitous, in that relinquishing control is not inherently negative. For example interview 6 describes a sense of relief in letting others take over and a sense of needing to be cared for. This is mirrored in the tabled examples from the literature [39,41].

Green & Baston [53] and Jomeen [49] have highlighted how the context of caring is essential in the ceding of control for women in labour. Crossley [25] who goes on to criticise the process on reflection describes (with a sense of guilt) her original sense of relief at the ability to 'let things go'. A lot of women talk about putting themselves in others' 'hands' and the references to safety and risk pervade women's accounts. In other words for many of these women there is an inevitability about relinquishing control, and in some cases this is by no means negative. This may be linked to original choices women make about the type of birth experience they desire. Control, when removed or ceded unwillingly, as evidenced by Walker et al [30] is viewed as a much more negative experience.

There are clear aspects of coercion within the accounts even if women themselves are not consciously aware of it. Pain figures largely in these narratives as well as the foetocentric discourse. Hospitals control pain relief, which means that it cannot easily be obtained otherwise. Hospitals also promise the least risky option for a healthy baby. Viisainen [31] offers interesting insight into this wider social discourse in highlighting that most people do not want to step out of line, 'especially not Finns'. This is a particularly telling quote as her study is of people who are overtly assertive and autonomous. That they feel unable to easily break social mores highlights strongly the power of the dominant discourse.

## Discussion

The narrative themes identified here can be resolved into one complex narrative revealing the continued dominance of medical discourse within maternity care and its continued permeation of the culture of maternity care which in turn continues to subordinate women. The interaction of women's own feelings with the cultural norms of maternity care alongside their continued subordination to the maternal principle fostered by western patriarchal society presents an illuminating picture of women's contemporary childbirth experiences.

One of the difficulties of the choice premise is that it engenders responsibility for the choices made [54]. This is particularly pertinent in maternity care where the stakes are so high and women are explicitly bound by the consequences of making the wrong choices. Choices, it seems, are made with notions of control inherently embedded and the desire and/or willingness to relinquish control to experts is overwhelmingly lucid in women's accounts, irrespective of who that expert may be. So whilst women engage with expert accounts in many guises to inform choices and enhance their sense of control during labour and childbirth this often fails to translate to a level of personal expertise that can effectively challenge the dominant discourse. The extent to which this should be facilitated is similarly complex but is currently grounded in paternalistic benevolence as opposed to open discussion as evidenced by the experiences of women who challenge this dominant discourse by opting for a home birth. These findings reinforce Edwards' [5] claim that choice is constructed through pervading belief systems and as long as foetocentrism predominates the status quo is likely to persist, as a healthy baby trumps all previous violations [25] and the end justifies the means.

This point is not new. Early feminist writers such as Oakley [46] highlighted such tensions thirty years ago. Indeed the fact that the literature utilised in this study spans well over a decade suggests the reality of choice for women in childbirth continues to contest the rhetoric. However the original contribution of this paper is the identification of a generalisable process grounded in international literature. Global evidence has been gathered here and synthesised showing that positive experience in childbirth is related to the amount of control experienced by the mother. This control is individually experienced and constructed from local narratives grounded in the dominant discourse. If the dominant discourse offers choice then this can only be facilitated if it is a genuine choice. This paper has shown that control can subsequently be supported by skilled health professionals who respect and understand the importance of choice in the birth process and have the capacity to help. This is best articulated by Kennedy et al's [35] study of expert midwives:

... *impressing upon families that you are there for them while they're in labor. I think that's so essential, because if you set someone up to believe that this is possible, and there's no one there who can carry that out, [then] they're left in the hands of unskilled professionals who don't know how to facilitate normal birth, and that's not fair*. [[35], emphasis added]

A criticism levelled at qualitative research in general often pertains to issues of interpretation and bias in particular, even from its own protagonists [55]. Although this criticism confounds quality issues within paradigms it is widely agreed the sample, methods and analysis should be transparent in order to offset any such claims. One critique of the interview sample is that these women were managed at home as part of an RCT rather than making a choice for home management of labour. However, these women made a choice to take part in the study which implies they saw home management as an attractive option. This limitation is also offset by the incorporation of interview data from the literature which gave the opportunity to evidence these themes within a much wider sample. The 24 studies presented selected quotes from a total sample of 656 women.

The widening of the sample to offset claims of interpretive bias links to a different criticism that questions the coherence and desirability of generalisable qualitative research. Regarding coherence, in analysing ethnographic, grounded theory, phenomenological and narrative analytic data together it could be claimed the product is epistemologically inconsistent. However, the data analysed here was primary data as presented within those paradigms. Although different researchers may have chosen to present different narrative exemplars within their methodologically distinct papers, this does not prevent further analysis of those exemplars. There may have been narrative exemplars contradicting the interpretation here, but they were not published. Regarding the desirability of generalisable qualitative research, this depends on the purpose of the research. If the purpose of research is to inform clinical practice then raising the generalisability of the findings is the best way to influence policy, guidelines and practice [56]. If the purpose of the research is to be ontologically distinct then concurrent analysis may rightly be considered a pragmatic approach.

## Conclusions

The original study [1,19] found women to express higher levels of internal control when hospitalised following PROM. Whilst this appeared at odds with other literature in this field, the qualitative findings offered illuminating reasons as to why this might be the case. The women in the qualitative element of the study lucidly subscribed to the dominant discourse of hospital as the safest place to give birth, under the premise of assuring a live healthy baby irrespective of their management type. The integration of the interview narratives with other women's experiences, using concurrent analysis, establishes confidence in the original assumptions and interpretations made as well as offering a broader, richer and nuanced depiction of the complexity of women's experiences of choice and control in childbirth.

## Competing interests

The authors declare that they have no competing interests.

## Authors' contributions

AS developed the theoretical framework and drafted the manuscript. CM developed the theoretical framework, drafted the manuscript and conducted the original PROM study. JJ conducted the original PROM study and assisted in the critical revision of the manuscript. CHM drafted the manuscript, assisted in the critical revision of the manuscript and participated in the coordination of the study. All authors read and approved the final manuscript.

## Pre-publication history

The pre-publication history for this paper can be accessed here:

http://www.biomedcentral.com/1471-2393/11/40/prepub
